# Urinary Albumin-to-Creatinine Ratio in Normal Range, Cardiovascular Health, and All-Cause Mortality

**DOI:** 10.1001/jamanetworkopen.2023.48333

**Published:** 2023-12-19

**Authors:** Nayili Mahemuti, Jiao Zou, Chuanlang Liu, Zhiyi Xiao, Fengchao Liang, Xueli Yang

**Affiliations:** 1 Department of Occupational and Environmental Health, School of Public Health, Tianjin Medical University, Tianjin, China; 2 School of Public Health and Emergency Management, Southern University of Science and Technology, Shenzhen, China; 3 Key Laboratory of Prevention and Control of Major Diseases in the Population, Ministry of Education, Tianjin Medical University, Tianjin, China; 4 Tianjin Key Laboratory of Environment, Nutrition and Public Health, Tianjin Medical University, Tianjin, China

## Abstract

**Importance:**

Although cumulative evidence suggests that elevated urinary albumin-to-creatinine ratio (UACR) in the normal range (<30 mg/g) may be associated with an increased risk of mortality, few studies have investigated whether cardiovascular health (CVH) modifies the harmful outcomes of high-normal UACR.

**Objective:**

To investigate associations of traditionally normal UACR and CVH with all-cause mortality.

**Design, Setting, and Participants:**

This cohort study used National Health and Nutrition Examination Survey data from 2005 through 2018 and linked mortality information until 2019. Data were analyzed from March 1 through October 31, 2023. The study included adult participants aged 20 to 79 years with a normal UACR (<30 mg/g) based on Kidney Disease: Improving Global Outcomes criteria.

**Exposures:**

The UACR was treated as a continuous variable and categorized into tertiles delineated as low (<4.67 mg/g), medium (4.67-7.67 mg/g), and high (7.68 to <30 mg/g). Cardiovascular health was assessed using Life’s Essential 8 scores and grouped as poor (0-49 points), moderate (50-79 points), and ideal (80-100 points).

**Main Outcomes and Measures:**

Multivariable Cox proportional hazards regression was used to estimate hazard ratios (HRs) and 95% CIs for associations of UACR with all-cause mortality in total participants and as stratified by CVH groups.

**Results:**

The study included 23 697 participants (mean [SD] age, 45.58 [15.44] years; 11 806 women [49.7%] and 11 891 men [50.3%]). During the median 7.8 years (range, 4.5-11.1 years) of follow-up, 1403 deaths were recorded. Near-linear associations were observed for continuous UACR and CVH with all-cause mortality. Compared with the low UACR group, high UACR in the normal range showed an increased mortality risk in the moderate and poor CVH groups (CVH [50-79]: HR, 1.54 [95% CI, 1.26-1.89]; CVH [0-49]: HR, 1.56 [95% CI, 1.10-2.20]), with a significant multiplicative interaction of UACR and CVH (*P* < .001).

**Conclusions and Relevance:**

The findings suggest that high UACR within the normal range is associated with a significantly increased risk of all-cause mortality, with the association more pronounced in adults with poor CVH status. These findings highlight the importance of risk management for early kidney dysfunction, particularly among individuals with poor CVH.

## Key Points

**Question:**

Does cardiovascular health (CVH) modify the association of all-cause mortality with elevated urinary albumin-to-creatinine ratio (UACR) within the normal range (<30 mg/g)?

**Findings:**

In this cohort study of 23 697 US adults with a UACR less than 30 mg/g, the risk of all-cause mortality associated with a high-normal UACR gradually increased across CVH groups as delineated by Life’s Essential 8 scores. Participants with poor CVH status and high-normal UACR had the highest risk of all-cause mortality.

**Meaning:**

The findings suggest that maintaining ideal CVH may reduce the risk of all-cause mortality associated with high-normal UACR, highlighting the importance of risk management for early kidney dysfunction.

## Teaser

This cohort study investigates the association of a high urinary albumin-to-creatinine ratio within the normal range and cardiovascular health with all-cause mortality in US adults.

## Introduction

Chronic kidney disease (CKD) is a major public health issue and directly contributes to the global burden of incidence and mortality,^1,2^ yet CKD often goes undiagnosed due to a lack of apparent symptoms in the early stages.^3^ The Kidney Disease: Improving Global Outcomes guideline suggests that the urinary albumin-to-creatinine ratio (UACR), with its good specificity and sensitivity, plays a role in the early diagnosis of CKD, with a UACR of 30 mg/g or greater defined as kidney injury.^4^ However, several studies have shown that risks of hypertension, cardiovascular disease (CVD), and all-cause mortality increase with an elevated UACR, even within the normal range of less than 30 mg/g,^5,6,7^ suggesting the importance of identifying high-risk individuals susceptible to mildly raised UACR in the traditional normal range.

The American Heart Association has proposed cardiovascular health (CVH) to encourage a paradigm shift from a merely disease-focused approach toward one inclusive of positive health maintenance throughout the life course.^8^ Several studies have shown that achieving a higher CVH score based on Life’s Essential 8 adopted by American Heart Association may be correlated with a lower risk of premature CVD, CVD mortality, and all-cause mortality.^9,10^ In addition, components of CVH, such as smoking, inadequate physical activity, high body mass index, hypertension, and diabetes, are known to pose hazards with regard to CKD.^11^ Nevertheless, it remains unclear whether CVH metrics can influence associations of adverse health outcomes with mild kidney dysfunction, as indicated by raised UACR within the normal range (ie, <30 mg/g). Given that CVH metrics are crucial and modifiable risk factors for survival, it is essential to explore whether and how health risks associated with high-normal UACR might vary based on an individual’s CVH status. Such investigation could extend our understanding of the long-term influence of kidney dysfunction within the traditional normal UACR range and provide valuable insight into identifying high-risk populations that require targeted monitoring and interventions in the future. The aim of this study was to investigate the association of combined UACR in the normal range and CVH status with all-cause mortality among US adults and to further explore whether elevated UACR within the normal range could mediate the association between poor CVH and all-cause mortality risks.

## Methods

### Study Population

This cohort study uses data from 7 continuous cycles of the National Health and Nutrition Examination Survey (NHANES) from 2005 to 2018. The National Center for Health Statistics ethics review board authorized the NHANES, and all participants provided written informed consent.^12^ The study reported here used publicly available and deidentified data; thus, the institutional review board of Tianjin Medical University determined that this study was exempt from review and the need for informed consent. This study followed the Strengthening the Reporting of Observational Studies in Epidemiology (STROBE) reporting guideline.

The NHANES is a cross-sectional survey that collects data from a nationally representative sample of noninstitutionalized US citizens, using a stratified, multistage random sampling technique.^13^ Among 36 973 adults aged 20 to 79 years, we excluded those (1) with missing CVH scores (n = 6744), (2) without information on urine albumin and creatinine levels or estimated glomerular filtration rate (eGFR) (n = 305), (3) with a UACR of 30 mg/g or greater (n = 3270), (4) with missing data on potential covariates (n = 2236), (5) who were pregnant or breastfeeding (n = 695), and (6) with unclear vital status due to being ineligible for mortality record linkage (n = 26). An analytic sample of 23 697 participants was included in this study (eFigure 1 in Supplement 1).

### Definition of UACR

Participants supplied urine specimens at the time of survey completion, and frozen urine samples (−20 °C) were sent to a Clinical Laboratory Improvement Amendments–certified laboratory. The NHANES website provides a complete summary of the laboratory methodology.^14^

Urine albumin and creatinine levels were used to compute the UACR. Urine albumin level reported in NHANES was measured using a solid phase fluorescent immunoassay. Urine creatinine level was assessed using the kinetic Jaffe rate reaction method before 2007 and the enzymatic method after 2007. Thus, we used the NHANES- recommended equation to adjust urine creatinine levels from the 2005-2006 cycles.^15^ In addition to treating UACR as a continuous variable, the UACRs within the normal range were further categorized into tertiles of low (<4.67 mg/g), medium (4.67-7.67 mg/g), and high (7.68 to <30 mg/g) for this study.

### Definitions of CVH Scores in Life’s Essential 8

The calculation of the Life’s Essential 8 CVH score is based on 8 components: diet quality, physical activity duration, smoking status, sleep duration, body mass index, blood lipids, blood glucose, and blood pressure.^8^ Each of these 8 components has its own set of scoring standards ranging from 0 to 100.^8^ Information on collection and measurements for the components of CVH are shown in the eMethods in Supplement 1, and the thresholds and specifics of the scoring are described in detail in eTable 1 in Supplement 1. The total CVH score was derived as the mean of the 8 metrics and further categorized into poor (0-49 points), moderate (50-79 points), and ideal (80-100 points) groups.^8^

### Outcomes and Covariates

The main outcome of the study was all-cause mortality. Death data were obtained by linking the cohort database with the National Death Index until December 31, 2019. Time to event was calculated from the NHANES examination day to the death date or the end of follow-up (December 31, 2019), whichever occurred first.

In this study, covariates included age, sex, self-reported race and ethnicity (Hispanic, non-Hispanic Black, non-Hispanic White, and other [including American Indian or Alaska Native, Asian, Native Hawaiian or Pacific Islander, multiple races or ethnicities, or unknown]), educational level (less than 9th grade, 9th-11th grade, some college or an associate’s degree, and college graduate or above), marital status (married and unmarried), poverty income ratio, heart disease (yes and no), stroke (yes and no), cancer (yes and no), and eGFR as calculated using the CKD Epidemiology Collaboration Creatinine Equation.^16^ For race and ethnicity, the NHANES first asked participants whether they were of Hispanic (including Mexican and non-Mexican Hispanic), Latino, or Spanish ancestry. Participants then were asked about their race, as categorized by NHANES. Per NHANES analytic guidelines, all participants self-reporting Hispanic ethnicity were categorized as Hispanic, regardless of race, while participants self-reporting as non-Hispanic were categorized according to their race.^17,18^ Previous studies using NHANES data reported racial disparities in cardiovascular health metrics, such as obesity, diabetes, physical inactivity, and serum creatinine.^17,19,20,21^ Thus, we included the race and ethnicity variable as a potential confounder.

### Statistical Analysis

Data analysis was conducted between March 1 and October 31, 2023. Given the NHANES complex sampling method, all analyses in this study applied sample weights, clustering, and stratification.^22^ We estimated the distribution of baseline and CVH factors based on UACR groups. Categorical variables are reported as percentages and continuous variables as means with SDs. Comparisons of baseline characteristics across each UACR group were conducted using χ^2^ tests for categorical variables and analysis of variance for continuous variables.

Multivariable Cox proportional hazards regression models were used to estimate hazard ratios (HRs) and 95% CIs of all-cause mortality associated with UACR as a continuous variable (per 10 mg/g increment) and category variable (tertiles of UACR) in the total sample and as stratified by CVH scores. The proportional hazards assumption was tested using Schoenfeld residuals. Model 1 was adjusted for age, sex, race and ethnicity, education level, marital status, poverty income ratio, and eGFR. Model 2 was additionally adjusted for history of heart disease, stroke, and cancer. The associations of tertiles of UACR with all-cause mortality were examined for each CVH group, using the lowest tertile as the reference group. To investigate the multiplicative interaction of the CVH and UACR groups, a cross-product term was introduced to the Cox regression model, and the multiplicative interaction effect was determined if *P* < .05 for the interaction term. Furthermore, we created a 9-category variable to represent joint exposures combining tertiles of UACR (<4.67, 4.67-7.67, and 7.68 to <30 mg/g) and CVH groups (0-49, 50-79, and 80-100 points) and performed the trend test to estimate joint effects by using the participants with both the ideal CVH (80-100 points) and the low UACR (<4.67 mg/g) as the reference. In addition, restricted cubic spline models with 3 knots (10th, 50th, and 90th percentiles) were fitted to investigate nonlinear associations of continuous UACRs and CVH scores with all-cause mortality. The likelihood ratio test was performed for the nonlinearity testing.

In subgroup analyses, we repeated the main analyses stratified by sex, age (<60 years vs ≥60 years), hypertension (yes vs no), and diabetes (yes vs no) using the continuous variable of UACR. We also conducted several sensitivity analyses to test the robustness of the main results. First, the CVH metrics in Life’s Essential 8 were added into the regression analysis as covariates. Second, participants with eGFR less than 60 mL/min/1.73 m^2^ were further excluded from the main analyses. Third, we excluded participants who died during the first 2 years of follow-up to reduce bias due to reverse causality. Fourth, we further adjusted for hypertension (yes or no) and diabetes (yes or no) in addition to covariates in model 2 to examine whether the risk factors would drive a substantial proportion of mortality risk associated with UACR.

In mediation analyses, we examined the extent to which an increment in UACR serves as a mediator for the association between CVH (ie, the ideal CVH group [80-100 points] as the reference) and all-cause mortality, adjusting for covariates in model 2. The approach of causal mediation analysis with survival data was used, and more details on the methodology of causal effect estimation under the counterfactual framework are described elsewhere.^23^

The statistical analysis was performed using SAS, version 9.4 (SAS Institute) and R, version 4.2.2 (R Foundation for Statistical Computing) software. All hypothesis tests were 2-sided, and *P* < .05 was considered statistically significant.

## Results

The analytic sample included 23 697 participants (mean [SD] age, 45.58 [15.44] years; 11 806 women [49.7%] and 11 891 men [50.3%]; 4841 Black [10.0%], 5969 Hispanic [13.3%], 10 344 White [69.7%], and 2543 other race and ethnicity [7.1%]). Detailed participant demographics according to tertiles of weighted UACRs (low [<4.67 mg/g], medium [4.67-7.67 mg/g], and high [7.68 to <30 mg/g]) are shown in Table 1. Compared with the low UACR tertile, participants in the high UACR tertile had higher percentages of hypertension; diabetes; and history of heart disease, stroke, and cancer. For the CVH assessment, more participants had poor CVH scores (0-49 points) in the high UACR tertile. Detailed CVH metrics of the study population are shown in eTable 2 in Supplement 1. There were slight differences in several baseline characteristics, such as sex and education levels, between the included participants and those excluded due to missing CVH data (eTable 3 in Supplement 1).

**Table 1.** Baseline Characteristics Based on Urinary Albumin-to-Creatinine Ratio (UACR) Tertiles in the National Health and Nutrition Examination Survey, 2005-2018^a^

Characteristic	No. (weighted %)				*P* value
	Total (n = 23 697)	UACR, mg/g^b^			
		Low (n = 7336)	Medium (n = 7604)	High (n = 8757)	
Age, mean (SD), y	45.58 (15.44)	42.26 (14.45)	45.70 (15.07)	48.71 (16.06)	<.001
Sex					
Male	11 891 (50.3)	4887 (67.1)	3529 (45.7)	3475 (38.3)	<.001
Female	11 806 (49.7)	2449 (32.9)	4075 (54.3)	5282 (61.7)	
Race and ethnicity					
Black	4841 (10.0)	1793 (11.8)	1360 (8.5)	1688 (9.6)	
Hispanic	5969 (13.3)	1566 (12.0)	1985 (13.4)	2418 (14.5)	<.001
White	10 344 (69.7)	3207 (69.4)	3408 (70.6)	3729 (69.0)	
Other^c^	2543 (7.1)	770 (6.9)	851 (7.5)	922 (6.9)	
Education level					
<9th Grade	2023 (4.2)	487 (3.5)	617 (4.0)	919 (5.1)	<.001
9th-11th Grade	3136 (9.6)	875 (8.4)	1014 (9.7)	1247 (10.7)	
Some college or associate’s degree	12 664 (54.9)	3970 (54.6)	3981 (53.1)	4713 (56.9)	
College graduate or above	5874 (31.3)	2004 (33.5)	1992 (33.2)	1878 (27.3)	
Marital status					
Married	12 474 (56.8)	3907 (57.7)	4036 (57.1)	4531 (55.7)	.20
Unmarried	11 223 (43.2)	3429 (42.3)	3568 (42.9)	4226 (44.3)	
Poverty income ratio, mean (SD), %	3.12 (1.64)	3.24 (1.63)	3.15 (1.63)	2.96 (1.64)	<.001
Hypertension	8304 (31.2)	1778 (22.2)	2496 (29.7)	4030 (41.4)	<.001
Diabetes	2847 (8.8)	489 (4.7)	765 (7.7)	1593 (14.0)	<.001
History					
Heart disease	1202 (4.4)	269 (3.1)	325 (3.8)	608 (6.1)	<.001
Stroke	610 (1.9)	114 (1.2)	190 (1.9)	306 (2.8)	<.001
Cancer	1811 (8.6)	391 (6.2)	568 (8.6)	852 (11.1)	<.001
eGFR, mean (SD), mL/min/1.73 m^2^	95.43 (19.78)	94.70 (19.02)	96.64 (19.13)	94.96 (21.04)	<.001
Total CVH scores, mean (SD)	65.89 (14.9)	67.69 (14.29)	66.34 (14.86)	63.69 (15.45)	<.001
CVH scores					
80-100	3797 (19.4)	1388 (21.9)	1248 (19.9)	1125 (16.5)	<.001
50-79	15 686 (65.5)	4973 (66.7)	5079 (66.4)	5634 (63.5)	
0-49	4214 (15.1)	975 (11.4)	1241 (13.6)	1998 (20.0)	

Abbreviations: CVH, cardiovascular health; eGFR, estimated glomerular filtration rate; UACR, urinary albumin-to-creatinine ratio.

^a^ Data are weighted to account for complex survey designs.

^b^ Grouped by tertiles into low (<4.67 mg/g), medium (4.67-7.67 mg/g), high (7.68 to <30 mg/g).

^c^ Other includes American Indian or Alaska Native, Asian, Native Hawaiian or Pacific Islander, multiple races or ethnicities, or unknown.

### All-Cause Mortality Associated With CVH Scores

**Figure 1.**
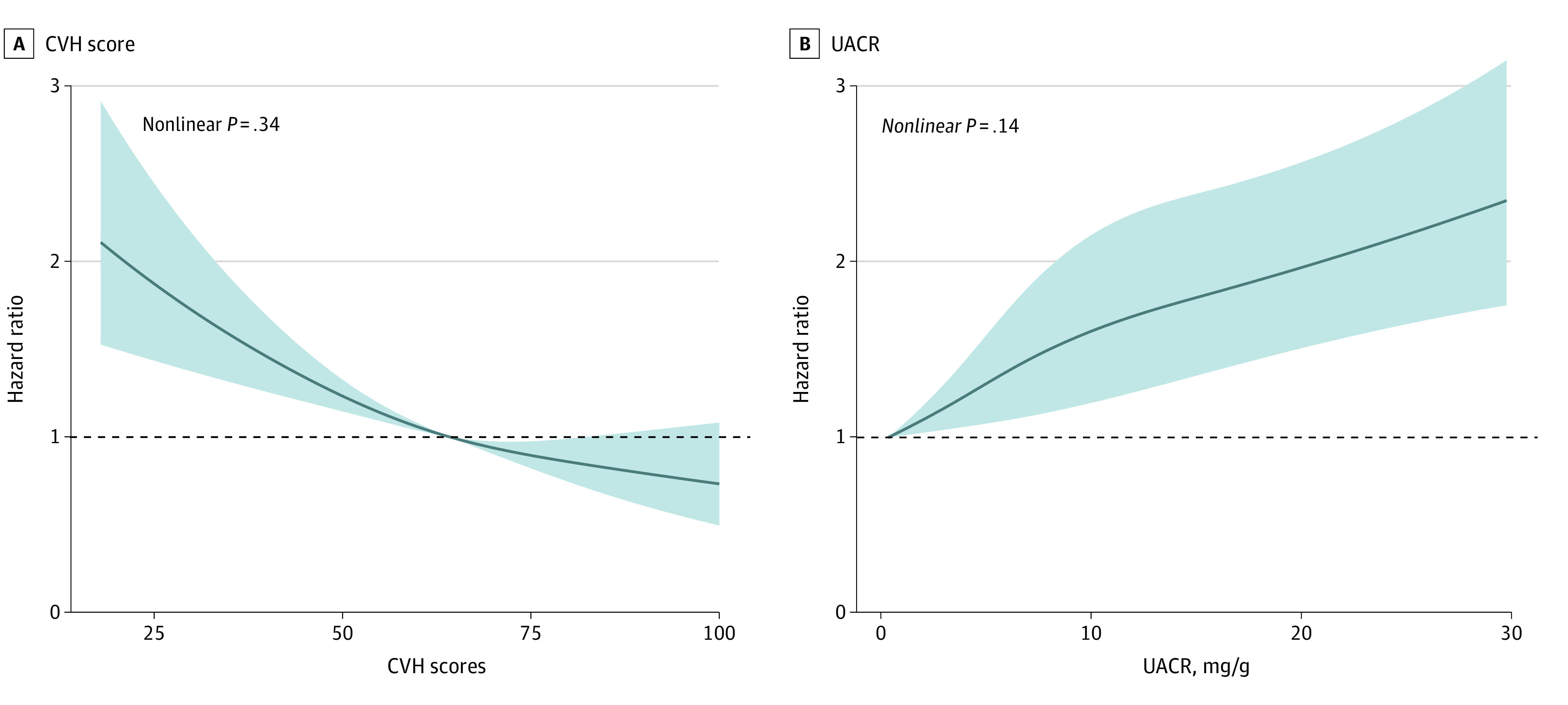
Association of Cardiovascular Health (CVH) Scores and Urinary Albumin-to-Creatinine Ratios (UACRs) With All-Cause Mortality Using Restricted Cubic Spline Models Hazard ratios (solid lines) and 95% CIs (shaded areas) were estimated after adjusting for age; sex; race and ethnicity; education level; marital status; poverty income ratio; estimated glomerular filtration rate; and history of heart disease, stroke, and cancer. The restricted cubic spline regression models were conducted with 3 knots at the 10th, 50th, and 90th percentiles of CVH score and UACR.

Over the median follow-up of 7.8 years (range, 4.5-11.1 years), a total of 1403 deaths were recorded among the study participants within the traditional normal range of UACR (<30 mg/g). Restricted cubic spline modeling showed a near-linear correlation between increased CVH score and reduced risk of all-cause mortality (Figure 1A). Compared with the ideal CVH (80-100) group, the HRs of all-cause mortality were 1.23 (95% CI, 0.94-1.60) and 1.71 (95% CI, 1.27-2.32) in the moderate CVH (50-79) and poor CVH (0-49) groups, respectively (eTable 4 in Supplement 1).

### All-Cause Mortality Associated With Continuous UACR Measurement in Different CVH Groups

Using restricted cubic spline modeling, a near-linear correlation was found between UACR and risk of all-cause mortality (Figure 1B). Table 2 shows HRs (95% CIs) for all the participants and each CVH group. All participants had an elevated risk of all-cause mortality, with an HR of 1.31 (95% CI, 1.20-1.44) per 10 mg/g increment in UACR after multivariable adjustment in model 2. Furthermore, a risk gradient of the UACR-mortality association was noted across CVH groups (*P* < .001 for trend), with corresponding HRs of 0.90 (95% CI, 0.59-1.39), 1.31 (95% CI, 1.19-1.44), and 1.38 (95% CI, 1.17-1.63) for the ideal, moderate, and poor CVH groups, respectively.

**Table 2.** Associations of All-Cause Mortality With Each 10 mg/g Increase of Urinary Albumin-to-Creatinine Ratio Among All the Participants and Those Stratified by Cardiovascular Health (CVH) Group

Model	HR (95% CI)			
	Total participants	CVH 80-100	CVH 50-79	CVH 0-49
No. of deaths (total No. of participants)	1403 (23 697)	83 (3797)	872 (15 686)	448 (4214)
Crude model^a^	1.77 (1.62-1.93)	1.23 (0.86-1.76)	1.77 (1.60-1.95)	1.55 (1.32-1.83)
Model 1^b^	1.34 (1.22-1.47)	0.92 (0.61-1.39)	1.33 (1.21-1.47)	1.40 (1.18-1.66)
Model 2^c^	1.31 (1.20-1.44)	0.90 (0.59-1.39)	1.31 (1.19-1.44)	1.38 (1.17-1.63)

Abbreviation: HR, hazard ratio.

^a^ No covariates were adjusted.

^b^ Adjusted for age, sex, race and ethnicity, education level, marital status, poverty income ratio, and estimated glomerular filtration rate.

^c^ Adjusted for age; sex; race and ethnicity; education level; marital status; poverty income ratio; estimated glomerular filtration rate; and history of heart disease, stroke, and cancer.

### Combined Association of UACR and CVH Status With All-Cause Mortality

Taking the tertiles of UACR as a categorical variable, Cox regression analyses in model 2 revealed that compared with the low UACR tertile, the high UACR tertile was associated with an increased risk of all-cause mortality among patients in the moderate and poor CVH groups, with HRs of 1.54 (95% CI, 1.26-1.89) and 1.56 (95% CI, 1.10-2.20), respectively (Table 3), with significant multiplicative interaction observed between UACR tertiles and CVH groups (*P* for interaction <.001).

**Table 3.** Associations of Tertiles of Urinary Albumin-to-Creatinine Ratio (UACR) With All-Cause Mortality Stratified by Cardiovascular Health (CVH) Group

Subgroup^a^	No. of deaths (No. of participants)	HR (95% CI)		
		Crude model^b^	Model 1^c^	Model 2^d^
CVH 80-100				
UACR low	21 (1388)	1 [Reference]	1 [Reference]	1 [Reference]
UACR medium	28 (1284)	0.96 (0.51-1.79)	0.73 (0.39-1.37)	0.72 (0.37-1.41)
UACR high	34 (1125)	1.59 (0.90-2.81)	1.09 (0.52-2.25)	1.06 (0.50-2.24)
CVH 50-79				
UACR low	187 (4973)	1 [Reference]	1 [Reference]	1 [Reference]
UACR medium	240 (5079)	1.55 (1.22-1.97)	1.32 (1.02-1.71)	1.32 (1.02-1.72)
UACR high	445 (5634)	2.52 (2.08-3.04)	1.59 (1.30-1.95)	1.54 (1.26-1.89)
CVH 0-49				
UACR low	79 (975)	1 [Reference]	1 [Reference]	1 [Reference]
UACR medium	104 (1241)	1.17 (0.77-1.77)	1.13 (0.75-1.71)	1.13 (0.74-1.72)
UACR high	265 (1998)	1.85 (1.32-2.60)	1.58 (1.11-2.25)	1.56 (1.10-2.20)

Abbreviation: HR, hazard ratio.

^a^ Grouped by tertiles into low (<4.67 mg/g), medium (4.67-7.67 mg/g), high (7.68 to <30 mg/g).

^b^ No covariates were adjusted.

^c^ Adjusted for age, sex, race and ethnicity, education level, marital status, poverty income ratio, and estimated glomerular filtration rate.

^d^ Adjusted for age; sex; race and ethnicity; education level; marital status; poverty income ratio; estimated glomerular filtration rate; and history of heart disease, stroke, and cancer.

**Figure 2.**
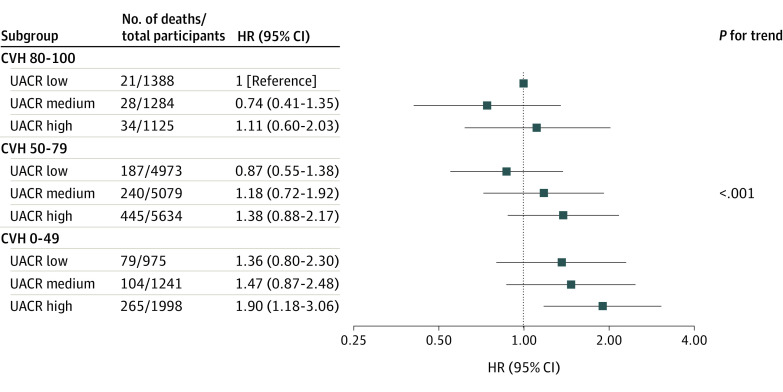
Joint Effect Analysis of Urinary Albumin-to-Creatinine Ratio (UACR) and Cardiovascular Health (CVH) Groups With Risks of All-Cause Mortality The multivariable Cox regression model was adjusted for age; sex; race and ethnicity; education level; marital status; poverty income ratio; estimated glomerular filtration rate; and history of heart disease, stroke, and cancer. HR indicates hazard ratio.

The potential joint effects of UACR and CVH on all-cause mortality were further examined after combining the UACR tertiles and CVH groups into a 9-category variable to represent the joint exposures. Among the various combinations, particularly in the CVH 0 to 79 range, participants with higher UACRs and poorer CVH status exhibited an increased risk of all-cause mortality (*P* < .001 for trend), despite nonsignificant HRs within most groups, which may be attributable to extensive categorization (Figure 2). Compared with both low UACR tertile and ideal CVH (80-100) group, participants in the high UACR tertile and poor CVH group had the highest risk of all-cause mortality (HR, 1.90; 95% CI, 1.18-3.06) (Figure 2).

### Subgroup and Sensitivity Analyses

Subgroup analyses by sex (male vs female), age (<60 years vs ≥60 years), hypertension (yes vs no), and diabetes (yes vs no) revealed similar patterns for associations between continuous UACR measurements and all-cause mortality across the CVH groups (eTables 5-8 in Supplement 1). In the sensitivity analyses, the results were similar to the main result when we fully adjusted all components of Life’s Essential 8 metrics as covariates (eTable 9 in Supplement 1), excluding participants with eGFR of less than 60 mL/min/1.73 m^2^ (eTable 10 in Supplement 1) and death during the first 2 years of follow-up, with participants in the poor CVH group still having the highest risk of mortality (HR, 1.37; 95% CI, 1.18-1.58) per 10 mg/g UACR increment (eTable 11 in Supplement 1). In addition, the multivariable regression analyses were conducted again after additional adjustment for hypertension and diabetes. The results were robust for both the continuous analysis stratified by CVH group (*P* < .001 for trend) (eTable 12 in Supplement 1) and the joint effects analysis based on the 9-category variables (*P* < .001 for trend) (eFigure 2 in Supplement 1).

### Mediation of CVH and All-Cause Mortality Associations Through UACR

We estimated that UACR mediated 7.8% and 10.5% of the associations of the moderate CVH (50-79) and poor CVH (0-49) groups, respectively, with all-cause mortality (eTable 13 in Supplement 1). For example, the indirect association of poor CVH via UACR indicated that we would, on average, observe a 5.0% (HR, 1.05; 95% CI, 1.03-1.07) increased risk of mortality in the poor CVH group compared with the ideal CVH (80-100) group. The proportion of the association between the poor CVH group and mortality mediated by UACR was 10.5%.

## Discussion

In this prospective cohort study, the risks of all-cause mortality were associated with an increased UACR and reduced CVH scores. An interaction of UACR and CVH on risk of all-cause mortality was observed. Compared with NHANES participants with both ideal CVH and low UACR, those with poor CVH and high UACR within the normal range had the highest risk of all-cause mortality. In addition, elevated UACR mediated 7.8% and 10.5% of the associations of moderate CVH and poor CVH, respectively, with all-cause mortality. These findings highlight the importance of risk management for early kidney dysfunction, particularly among individuals with poor CVH.

Several epidemiological studies have observed positive associations between UACR in the normal range and all-cause mortality.^5,7,24^ Inoue et al^5^ found that UACRs of both 10 to 30 mg/g and 5 to 10 mg/g may be linked to higher risks of all-cause mortality compared with UACRs of less than 5 mg/g. In the Strong Heart Study among American Indian participants, albuminuria levels with UACR less than 30 mg/g were also associated with an increased risk of CVD mortality.^24^ In this study, we used the updated data from NHANES 2005-2018 and found associations between high-normal UACR and all-cause mortality in participants stratified by different CVH scores, using both continuous measurement and tertiles of UACR. The associations of continuous UACR with all-cause mortality were significantly more pronounced across the ideal, moderate, and poor CVH groups, and a variety of sensitivity analyses showed that the results are robust. For example, participants who died within 2 years of follow-up were excluded to minimize potential reverse causation bias. The results showed that risks of all-cause mortality remained significant, increasing across the 3 CVH groups (*P* for trend <.001), and participants in the poor CVH group still had the highest risk of mortality (HR, 1.37; 95% CI, 1.18-1.58) per 10 mg/g UACR increment (eTable 11 in Supplement 1). Furthermore, we used tertiles of UACR and 3 CVH groups to create a 9-category variable to conduct joint effect analyses. A significant pattern of increasing mortality risks among the joint exposures was also observed, although statistical significance was not observed for some effect estimations due to the limited sample size within each stratum.

In addition, major risk factors for CVD, such as hypertension, dyslipidemia, hyperglycemia, and obesity, have been reported to be associated with new-onset kidney disease.^25^ The Framingham Offspring Study documented that the risk reduction of developing CKD and all-cause mortality later in life was closely associated with spending more time on improving CVH status.^26^ However, there is still insufficient evidence from cohort studies regarding CVH modification and associations between normal UACR and adverse events. In our study, we found that compared with the lowest tertile of UACR, HRs for all-cause mortality associated with the highest UACR tertile were 1.06 (95% CI, 0.50-2.24), 1.54 (95% CI, 1.26-1.89), and 1.56 (95% CI, 1.10-2.20) across the ideal, moderate, and poor CVH groups, respectively, with a significant multiplicative interaction effect between UACR and CVH groups observed for all-cause mortality (*P* < .001).

Moreover, to our knowledge, this study is the first to provide quantitative evidence that UACR may mediate the associations of CVH status with all-cause mortality in the general US population. The findings indicate that UACR elevation within the normal range may mediate 10.5% of the associations between poor CVH and all-cause mortality, which further supports the importance of kidney function evaluation, particularly among populations with high cardiovascular risk.

The specific biological mechanism underlying the deleterious outcomes of high-normal UACR is still unclear. Some research has indicated that the increased kidney endothelial permeability associated with microalbuminuria may be a sign of diffuse endothelial dysfunction, leading to cardiovascular damage and elevated risk of death.^27,28^ The UACR within the normal range or microalbuminuria may also induce alterations in von Willebrand factor, fibrinogen, and thrombomodulin.^29,30,31^ More investigation into the biological mechanisms of UACR and CVH associated with mortality are needed from population-based and animal studies.

### Strengths and Limitations

One of the key strengths of our study is the use of a nationally representative sample of US adults, which allows the findings to be generalized to a larger population. In addition, the detailed and high-quality data collected allowed us to control for well-known confounders, such as information on socioeconomics, lifestyle, and disease conditions.

Several limitations to our study should be considered. First, only 1 spot urine measurement from NHANES participants was used to assess urinary albumin excretion. Thus, the study results need to be interpreted with caution, considering the high variability of UACR reported in the traditional normal range (<30 mg/g).^32^ High variability of UACR in the normal range would make it difficult to distinguish random variability from meaningful biological changes. More studies with multiple urine measurements, preferably of the first morning void, may help with determining a threshold to estimate early kidney function damage. Second, self-reporting of the 4 health behavior factors of diet, physical activity, nicotine exposure, and sleep duration may result in recall bias. Third, the number of CVD-related deaths in each CVH group was relatively small, and we did not focus on CVD mortality. Fourth, although the risk threshold for normal UACR is not clear so far, several studies in American and Korean adults have observed risks of all-cause mortality or CVD outcomes associated with a high UACR in the normal range when the UACR is greater than 5 mg/g.^5,7,24^ More research is needed to explore safe thresholds for normal kidney function and to develop precise risk stratification combined with CVH information.

## Conclusions

In this cohort study of US adults, the findings show an association between high-normal UACR and all-cause mortality, particularly among those with suboptimal CVH, as indicated by a score of less than 80 points. Notably, CVH status modified risks of all-cause mortality associated with elevated UACRs in the conventional normal range (<30 mg/g). Although further validation among independent populations is warranted, these findings underscore the importance of early identification of high-risk populations with normal UACR values through assessment of CVH, which might be helpful to target risk interventions in the future.

## Notes

Supplement 1. **eMethods.** Information on Collection and Measurements for the Components of CVH **eTable 1.** Definition and Scoring Approach for the American Heart Association’s Life’s Essential 8 Score **eTable 2.** Cardiovascular Health Metrics Based on UACR Levels in NHANES 2005-2018 **eTable 3.** Comparisons of Baseline Characteristics Between Participants With Complete Data and Those With Missing CVH Data **eTable 4.** Adjusted Hazard Ratios and 95% CIs of CVH With Risk of All-Cause Mortality **eTable 5.** Adjusted Hazard Ratios and 95% CIs of UACR With Risk of All-Cause Mortality Stratified by Sex and CVH **eTable 6.** Adjusted Hazard Ratios and 95% CIs of UACR With Risk of All-Cause Mortality Stratified by Age and CVH **eTable 7.** Adjusted Hazard Ratios and 95% CIs of UACR With Risk of All-Cause Mortality Stratified by Hypertension and CVH **eTable 8.** Adjusted Hazard Ratios and 95% CIs of UACR With Risk of All-Cause Mortality Stratified by Diabetes and CVH **eTable 9.** Adjusted Hazard Ratios and 95% CIs of UACR With Risk of All-Cause Mortality in Models Further Adjusted for Covariates of CVH **eTable 10.** Adjusted Hazard Ratios and 95% CIs of UACR With Risk of All-Cause Mortality Stratified by CVH After Exclusion of Participants Whose eGFR was <60 mL/min/1.73 m^2^ **eTable 11.** Adjusted Hazard Ratios and 95% CIs of UACR With Risk of All-Cause Mortality Stratified by CVH After Excluding Death Within the First 2 Years of Follow-Up **eTable 12.** Adjusted Hazard Ratios and 95% CIs of UACR With Risk of All-Cause Mortality Stratified by CVH With Additional Adjustment for Hypertension and Diabetes **eTable 13.** Estimated Direct and Indirect Effect Sizes of Cardiovascular Health With All-Cause Mortality Through UACR in the National Health and Nutrition Examination Survey, 2005 to 2018 **eFigure 1.** Selection of Study Participants From the 2005 to 2018 Cycles of the National Health and Nutrition Examination Survey **eFigure 2.** HRs (95% CIs) for Associations of UACR With All-Cause Mortality Stratified by CVH Groups, After Adjusting for Hypertension and Diabetes
eReferences

Supplement 2.
Data Sharing Statement

## References

[1] Chronic kidney disease in the United States, 2023. Centers for Disease Control and Prevention. Accessed May 3, 2023. https://www.cdc.gov/kidneydisease/publications-resources/ckd-national-facts.html

[2] Jha V, Garcia-Garcia G, Iseki K, . Chronic kidney disease: global dimension and perspectives. Lancet. 2013;382(9888):260-272. doi:10.1016/S0140-6736(13)60687-X 23727169

[3] Lv JC, Zhang LX. Prevalence and disease burden of chronic kidney disease. Adv Exp Med Biol. 2019;1165:3-15. doi:10.1007/978-981-13-8871-2_1 31399958

[4] Levey AS, Eckardt KU, Tsukamoto Y, . Definition and classification of chronic kidney disease: a position statement from Kidney Disease: Improving Global Outcomes (KDIGO). Kidney Int. 2005;67(6):2089-2100. doi:10.1111/j.1523-1755.2005.00365.x 15882252

[5] Inoue K, Streja E, Tsujimoto T, Kobayashi H. Urinary albumin-to-creatinine ratio within normal range and all-cause or cardiovascular mortality among U.S. adults enrolled in the NHANES during 1999-2015. Ann Epidemiol. 2021;55:15-23. doi:10.1016/j.annepidem.2020.12.004 33338645 PMC8202057

[6] Kovesdy CP, Lott EH, Lu JL, . Outcomes associated with microalbuminuria: effect modification by chronic kidney disease. J Am Coll Cardiol. 2013;61(15):1626-1633. doi:10.1016/j.jacc.2012.11.071 23500283 PMC3625505

[7] Sung KC, Ryu S, Lee JY, . Urine albumin/creatinine ratio below 30 mg/g is a predictor of incident hypertension and cardiovascular mortality. J Am Heart Assoc. 2016;5(9):e003245. doi:10.1161/JAHA.116.003245 27625343 PMC5079007

[8] Lloyd-Jones DM, Allen NB, Anderson CAM, ; American Heart Association. Life’s Essential 8: updating and enhancing the American Heart Association’s construct of cardiovascular health: a presidential advisory from the American Heart Association. Circulation. 2022;146(5):e18-e43. doi:10.1161/CIR.0000000000001078 35766027 PMC10503546

[9] Sun J, Li Y, Zhao M, . Association of the American Heart Association’s new “Life’s Essential 8” with all-cause and cardiovascular disease-specific mortality: prospective cohort study. BMC Med. 2023;21(1):116. doi:10.1186/s12916-023-02824-8 36978123 PMC10053736

[10] Perak AM, Ning H, Khan SS, . Associations of late adolescent or young adult cardiovascular health with premature cardiovascular disease and mortality. J Am Coll Cardiol. 2020;76(23):2695-2707. doi:10.1016/j.jacc.2020.10.002 33181243 PMC8100998

[11] Vallianou NG, Mitesh S, Gkogkou A, Geladari E. Chronic kidney disease and cardiovascular disease: is there any relationship? Curr Cardiol Rev. 2019;15(1):55-63. doi:10.2174/1573403X14666180711124825 29992892 PMC6367692

[12] NCHS research ethics review (ERB) approval. Centers for Disease Control and Prevention. Accessed February 22, 2023. https://www.cdc.gov/nchs/nhanes/irba98.htm

[13] National Health and Nutrition Examination Survey. Centers for Disease Control and Prevention. Accessed February 25, 2023. https://www.cdc.gov/nchs/nhanes.htm

[14] National Health and Nutrition Examination Survey: serum, plasma, and urine specimens. Centers for Disease Control and Prevention. Accessed February 25, 2023. https://www.cdc.gov/nchs/nhanes/biospecimens/serum_plasma_urine.htm

[15] National Health and Nutrition Examination Survey: 2007-2008 data documentation, codebook, and frequencies. Centers for Disease Control and Prevention. Accessed February 25, 2023. https://wwwn.cdc.gov/Nchs/Nhanes/2007-2008/ALB_CR_E.htm#Laboratory_Quality_Assurance_and_Monitoring

[16] Levey AS, Stevens LA, Schmid CH, ; CKD-EPI (Chronic Kidney Disease Epidemiology Collaboration). A new equation to estimate glomerular filtration rate. Ann Intern Med. 2009;150(9):604-612. doi:10.7326/0003-4819-150-9-200905050-00006 19414839 PMC2763564

[17] Liu B, Du Y, Wu Y, Snetselaar LG, Wallace RB, Bao W. Trends in obesity and adiposity measures by race or ethnicity among adults in the United States 2011-18: population based study. BMJ. 2021;372:n365. doi:10.1136/bmj.n365 33727242 PMC7961695

[18] Yokose C, McCormick N, Lu N, . Trends in prevalence of gout among US Asian adults, 2011-2018. JAMA Netw Open. 2023;6(4):e239501. doi:10.1001/jamanetworkopen.2023.9501 37083663 PMC10122173

[19] Cheng YJ, Kanaya AM, Araneta MRG, . Prevalence of diabetes by race and ethnicity in the United States, 2011-2016. JAMA. 2019;322(24):2389-2398. doi:10.1001/jama.2019.19365 31860047 PMC6990660

[20] Yang L, Cao C, Kantor ED, . Trends in sedentary behavior among the US population, 2001-2016. JAMA. 2019;321(16):1587-1597. doi:10.1001/jama.2019.3636 31012934 PMC6487546

[21] Jain RB. Trends in the levels of urine and serum creatinine: data from NHANES 2001-2014. Environ Sci Pollut Res Int. 2017;24(11):10197-10204. doi:10.1007/s11356-017-8709-y 28265873

[22] Specifying weighting parameters. Centers for Disease Control and Prevention. Updated May 10, 2013. Accessed March 17, 2023. http://medbox.iiab.me/modules/en-cdc/www.cdc.gov/nchs/tutorials/nhanes/SurveyDesign/Weighting/intro.htm

[23] Li Y, Yoshida K, Kaufman JS, Mathur MB. A brief primer on conducting regression-based causal mediation analysis. Psychol Trauma. 2023;15(6):930-938. doi:10.1037/tra0001421 36701540 PMC10368791

[24] Xu J, Knowler WC, Devereux RB, . Albuminuria within the “normal” range and risk of cardiovascular disease and death in American Indians: the Strong Heart Study. Am J Kidney Dis. 2007;49(2):208-216. doi:10.1053/j.ajkd.2006.10.017 17261423

[25] Fox CS, Larson MG, Leip EP, Culleton B, Wilson PW, Levy D. Predictors of new-onset kidney disease in a community-based population. JAMA. 2004;291(7):844-850. doi:10.1001/jama.291.7.844 14970063

[26] Corlin L, Short MI, Vasan RS, Xanthakis V. Association of the duration of ideal cardiovascular health through adulthood with cardiometabolic outcomes and mortality in the Framingham Offspring Study. JAMA Cardiol. 2020;5(5):549-556. doi:10.1001/jamacardio.2020.0109 32159731 PMC7066529

[27] Stehouwer CD, Smulders YM. Microalbuminuria and risk for cardiovascular disease: analysis of potential mechanisms. J Am Soc Nephrol. 2006;17(8):2106-2111. doi:10.1681/ASN.2005121288 16825333

[28] Deckert T, Kofoed-Enevoldsen A, Nørgaard K, Borch-Johnsen K, Feldt-Rasmussen B, Jensen T. Microalbuminuria. implications for micro- and macrovascular disease. Diabetes Care. 1992;15(9):1181-1191. doi:10.2337/diacare.15.9.1181 1396015

[29] Agewall S, Fagerberg B, Attvall S, ; Risk Factor Intervention Study Group. Microalbuminuria, insulin sensitivity and haemostatic factors in non-diabetic treated hypertensive men. J Intern Med. 1995;237(2):195-203. doi:10.1111/j.1365-2796.1995.tb01161.x 7852923

[30] Kario K, Matsuo T, Kobayashi H, Matsuo M, Sakata T, Miyata T. Activation of tissue factor-induced coagulation and endothelial cell dysfunction in non-insulin-dependent diabetic patients with microalbuminuria. Arterioscler Thromb Vasc Biol. 1995;15(8):1114-1120. doi:10.1161/01.ATV.15.8.1114 7627704

[31] Hillege HL, Fidler V, Diercks GF, ; Prevention of Renal and Vascular End Stage Disease (PREVEND) Study Group. Urinary albumin excretion predicts cardiovascular and noncardiovascular mortality in general population. Circulation. 2002;106(14):1777-1782. doi:10.1161/01.CIR.0000031732.78052.81 12356629

[32] Coresh J, Selvin E, Stevens LA, . Prevalence of chronic kidney disease in the United States. JAMA. 2007;298(17):2038-2047. doi:10.1001/jama.298.17.2038 17986697

