# Supplementary material for: Urinary Albumin-to-Creatinine Ratio in Normal Range, Cardiovascular Health, and All-Cause Mortality
Source: JAMA Netw Open. 2023 Dec 19;6(12):e2348333. doi: 10.1001/jamanetworkopen.2023.48333 (PMC10731498; doi:10.1001/jamanetworkopen.2023.48333)
Supplement: Supplement 1. — eMethods. Information on Collection and Measurements for the Components of CVH eTable 1. Definition and Scoring Approach for the American Heart Association’s Life’s Essential 8 Score eTable 2. Cardiovascular Health Metrics Based on UACR Levels in NHANES 2005-2018 eTable 3. Comparisons of Baseline Characteristics Between Participants With Complete Data and Those With Missing CVH Data eTable 4. Adjusted Hazard Ratios and 95% CIs of CVH With Risk of All-Cause Mortality eTable 5. Adjusted Hazard Ratios and 95% CIs of UACR With Risk of All-Cause Mortality Stratified by Sex and CVH eTable 6. Adjusted Hazard Ratios and 95% CIs of UACR With Risk of All-Cause Mortality Stratified by Age and CVH eTable 7. Adjusted Hazard Ratios and 95% CIs of UACR With Risk of All-Cause Mortality Stratified by Hypertension and CVH eTable 8. Adjusted Hazard Ratios and 95% CIs of UACR With Risk of All-Cause Mortality Stratified by Diabetes and CVH eTable 9. Adjusted Hazard Ratios and 95% CIs of UACR With Risk of All-Cause Mortality in Models Further Adjusted for Covariates of CVH eTable 10. Adjusted Hazard Ratios and 95% CIs of UACR With Risk of All-Cause Mortality Stratified by CVH After Exclusion of Participants Whose eGFR was <60 mL/min/1.73 m2 eTable 11. Adjusted Hazard Ratios and 95% CIs of UACR With Risk of All-Cause Mortality Stratified by CVH After Excluding Death Within the First 2 Years of Follow-Up eTable 12. Adjusted Hazard Ratios and 95% CIs of UACR With Risk of All-Cause Mortality Stratified by CVH With Additional Adjustment for Hypertension and Diabetes eTable 13. Estimated Direct and Indirect Effect Sizes of Cardiovascular Health With All-Cause Mortality Through UACR in the National Health and Nutrition Examination Survey, 2005 to 2018 eFigure 1. Selection of Study Participants From the 2005 to 2018 Cycles of the National Health and Nutrition Examination Survey eFigure 2. HRs (95% CIs) for Associations of UACR With All-Cause Mortality Stratified by CVH Groups, After Adjusting fo [file jamanetwopen-e2348333-s001.pdf]

Supplemental Online Content

Mahemuti N, Zou J, Liu C, Xiao Z, Liang F, Yang X. Urinary albumin-to-creatinine ratio in normal range, cardiovascular health, and all-cause mortality. *JAMA Netw Open*. 2023;6(12):e2378333. doi:10.1001/jamanetworkopen.2023.48333

eMethods. Information on Collection and Measurements for the Components of CVH
eTable 1. Definition and Scoring Approach for the American Heart Association's Life's Essential 8 score
eTable 2. Cardiovascular Health Metrics Based on UACR Levels in NHANES 2005-2018
eTable 3. Comparisons of Baseline Characteristics Between Participants With Complete Data and Those With Missing CVH Data
eTable 4. Adjusted Hazard Ratios and 95% CIs of CVH With Risk of All-Cause Mortality
eTable 5. Adjusted Hazard Ratios and 95% CIs of UACR With Risk of All-Cause Mortality Stratified by Sex and CVH
eTable 6. Adjusted Hazard Ratios and 95% CIs of UACR With Risk of All-Cause Mortality Stratified by Age and CVH
eTable 7. Adjusted Hazard Ratios and 95% CIs of UACR With Risk of All-Cause Mortality Stratified by Hypertension and CVH
eTable 8. Adjusted Hazard Ratios and 95% CIs of UACR With Risk of All-Cause Mortality Stratified by Diabetes and CVH
eTable 9. Adjusted Hazard Ratios and 95% CIs of UACR With Risk of All-Cause Mortality in Models Further Adjusted for Covariates of CVH
eTable 10. Adjusted Hazard Ratios and 95% CIs of UACR With Risk of All-Cause Mortality Stratified by CVH After Exclusion of Participants Whose eGFR Was < 60mL/min/1.73 m^2
eTable 11. Adjusted Hazard Ratios and 95% CIs of UACR With Risk of All-Cause Mortality Stratified by CVH After Excluding Death Within the First 2 years of Follow-Up
eTable 12. Adjusted Hazard Ratios and 95% CIs of UACR With Risk of All-Cause Mortality Stratified by CVH With Additional Adjustment for Hypertension and Diabetes
eTable 13. Estimated Direct and Indirect Effect Sizes of Cardiovascular Health With All-Cause Mortality Through UACR in the National Health and Nutrition Examination Survey, 2005 to 2018
eFigure 1. Selection of Study Participants From the 2005 to 2018 Cycles of the National Health and Nutrition Examination Survey
eFigure 2. HRs (95% CIs) for Associations of UACR With All-Cause Mortality Stratified by CVH Groups, After Adjusting for Hypertension and Diabetes.
eReferences

This supplemental material has been provided by the authors to give readers additional information about their work.

© 2023 Mahemuti N et al. *JAMA Network Open.*

eMethods. Information on Collection and Measurements for the Components of CVH

Diet quality was assessed by the Healthy Eating Index 2015 (HEI-2015), and the data for Diet quality assessment came from the "Food Patterns Equivalents Database 14".[1] Physical activity was calculated by self-reported moderate or greater activity frequency per week and duration. According to the AHA, each minute of moderate exercise counts as one unit, while each minute of vigorous activity counts as two units.[2] Nicotine exposure was determined based on self-reported consumption of any nicotine product in the past 30 days, and secondhand nicotine exposure in a participant's household. Sleep duration was the average sleep duration per day. Body mass index was determined as weight (kg) divided by height squared (m^2).

Blood pressure was assessed by three consecutive times of systolic and diastolic measurements and the use of antihypertensive drugs. Blood samples were obtained at a mobile examination center and transmitted to a central laboratory to analyze plasma total and high-density lipoprotein (HDL) cholesterol, fasting blood glucose (FBG), and haemoglobin A1c (HbA1c). Lipid was assessed by plasma total and HDL cholesterol as well as the use of lipid-lowering medications. Blood glucose was assessed by FBG or HbA1c or history of diabetes.

© 2023 Mahemuti N et al. *JAMA Network Open.*

eTable 1. Definition and Scoring Approach for the American Heart Association's Life's Essential 8 Score [2]

CVH Metric	Method of measurement	Quantification of CVH Metric
Diet score	Healthy Eating Index-2015 diet score percentile.	Quantiles of Healthy Eating Index-2015<br><br>**Scoring (Population):**<br>Points Quantile<br>100 ≥95th percentile (top/ideal diet)<br>80 75th – 94th percentile<br>50 50th – 74th percentile<br>25 25th – 49th percentile<br>0 1st – 24th percentile (bottom/least ideal quartile)
Physical activity	Self-reported minutes of moderate or vigorous physical activity per week.1 minute of vigorous physical activity is equivalent to 2 minutes of moderate physical activity.	**Metric:** Minutes of moderate (or vigorous) intensity activity per week<br><br>**Scoring:**<br>Points Minutes<br>100 ≥150<br>90 120 – 149<br>80 90 – 119<br>60 60 – 89<br>40 30 – 59<br>20 1 – 29<br>0 0
Nicotine exposure	Self-reported use of cigarettes or inhaled nicotine- delivery system	**Metric:** Combustible tobacco use and/or inhaled NDS use; or secondhand smoke exposure<br><br>**Scoring:**<br>Points Status<br>100 Never smoker<br>75 Former smoker, quit ≥5 yrs<br>50 Former smoker, quit 1–<5 yrs<br>20 Former smoker, quit <1 year, or currently using inhaled NDS<br>0 Current smoker<br><br>Subtract 20 points (unless score is 0) for living with active indoor smoker in home

© 2023 Mahemuti N et al. *JAMA Network Open.*

CVH Metric	Method of measurement	Quantification of CVH Metric
Sleep health	Self-reported average hours of sleep per night	**Metric:** Average hours of sleep per night<br><br>**Scoring:**<br><u>Points</u> <u>Level</u><br>100 7 – <9<br>90 9 – <10<br>70 6 – <7<br>40 5 – <6 or ≥10<br>20 4 – <5<br>0 <4
Body mass index	Body weight (kg) divided by height squared (m^2)	**Metric:** Body mass index (kg/m^2)<br><br>**Scoring:**<br><u>Points</u> <u>Level</u><br>100 <25<br>70 25.0 – 29.9<br>30 30.0 – 34.9<br>15 35.0 – 39.9<br>0 ≥40.0
Blood lipids	Plasma total and HDL-cholesterol with calculation of non-HDL-cholesterol	**Metric:** Non-HDL-cholesterol (mg/dL)<br><br>**Scoring:**<br><u>Points</u> <u>Level</u><br>100 <130<br>60 130 – 159<br>40 160 – 189<br>20 190 – 219<br>0 ≥220<br><br>If drug-treated level, subtract 20 points

© 2023 Mahemuti N et al. *JAMA Network Open.*

CVH Metric	Method of measurement	Quantification of CVH Metric
Blood glucose	Fasting blood glucose or casual hemoglobin A1c	**Metric:** Fasting blood glucose (mg/dL) or Hemoglobin A1c (%)<br><br>**Scoring:**<br><u>Points</u> <u>Level</u><br>100 No history of diabetes and FBG <100 (or HbA1c < 5.7)<br>60 No diabetes and FBG 100 – 125 (or HbA1c 5.7 – 6.4) (Prediabetes)<br>40 Diabetes with HbA1c <7.0<br>30 Diabetes with HbA1c 7.0 – 7.9<br>20 Diabetes with HbA1c 8.0 – 8.9<br>10 Diabetes with HbA1c 9.0 – 9.9<br>0 Diabetes with HbA1c ≥10.0
Blood pressure	Appropriately measured systolic and diastolic blood pressure	**Metric:** Systolic and diastolic blood pressure (mm Hg)<br><br>**Scoring:**<br><u>Points</u> <u>Level</u><br>100 <120/<80 (Optimal)<br>75 120 – 129/<80 (Elevated)<br>50 130 – 139 or 80 – 89 (Stage I HTN)<br>25 140 – 159 or 90 – 99<br>0 ≥160 or ≥100<br><br>Subtract 20 points if treated level

Abbreviation: CVH, cardiovascular health; NDS, nicotine-delivery systems; HDL, high-density lipoprotein; FBG, fasting blood glucose; HbA1c, glycated hemoglobin; HTN, hypertension.

Note: This table is adapted from Life's Essential 8: Updating and Enhancing the American Heart Association's Construct of Cardiovascular Health: A Presidential Advisory From the American Heart Association.[2]

© 2023 Mahemuti N et al. *JAMA Network Open.*

eTable 2. Cardiovascular Health Metrics Based on UACR Levels in NHANES 2005-2018[a]

Characteristic	UACR Low (<4.67 mg/g) N=7336	UACR Medium (4.67 to 7.67 mg/g) N=7604	UACR High (7.68 to <30.0 mg/g) N=8757	*P*-value
**Diet**				
Mean score (SD)	41.17 (31.30)	42.70 (31.48)	43.08 (31.33)	<.001
**Physical activity**				
Mean score (SD)	51.67 (46.56)	46.36 (46.20)	40.48 (45.54)	<.001
**Nicotine exposure**				
Mean score (SD)	67.67 (38.03)	68.82 (37.62)	68.49 (37.67)	.16
**Sleep health**				
Mean score (SD)	81.70 (24.89)	81.41 (25.52)	80.73 (25.97)	.03
**Body mass index**				
Mean score (SD)	60.67 (32.40)	60.80 (33.68)	58.04 (34.54)	<.001
**Blood lipids[b]**				
Mean score (SD)	66.17 (30.24)	64.53 (30.95)	64.05 (31.08)	<.001
**Blood glucose**				
Mean score (SD)	87.30 (21.06)	84.37 (23.65)	78.44 (28.09)	<.001
**Blood pressure**				
Mean score (SD)	73.60 (28.41)	68.40 (30.68)	60.10 (32.96)	<.001

Abbreviation: UACR, urinary albumin-to-creatinine ratio.

[a] Data were presented as weighted mean (SD).

[b] Blood lipids used the level of non-HDL cholesterol according to the AHA definitions of Life's Essential 8.

© 2023 Mahemuti N et al. *JAMA Network Open.*

eTable 3. Comparisons of Baseline Characteristics Between Participants With Complete Data and Those With Missing CVH Data

Characteristic	No. (%) of participants[a]		*P*-value
	Participants with complete data (N=23697)	Participants with missing CVH data (N=6744)	
**Age, mean (SD), y**	46.73 (16.10)	47.28 (16.85)	.02
**Sex**			
Male	11896 (50.2)	3210 (47.6)	< .001
Female	11806 (49.8)	3234 (52.4)	
**Race and ethnicity**			
Hispanic	5969 (25.2)	1695 (25.2)	< .001
White	10344 (43.7)	2030 (30.1)	
Black	4841 (20.4)	1849 (27.4)	
Other[b]	2543 (10.7)	1170 (17.3)	
**Education level**			
< 9th grade	2023 (8.5)	891 (13.2)	< .001
9th-11th grade	3136 (13.2)	1075 (15.9)	
Some college or associate's degree	12664 (53.4)	3364 (49.9)	
College graduate or above	5874 (24.8)	1392 (20.6)	
**Marital status**			
Married	12474 (52.7)	3223 (47.8)	< .001
Unmarried	11223 (47.3)	3505 (52.0)	
**Poverty income ratio, mean (SD)**	2.61 (1.65)	2.31 (1.62)	< .001

Abbreviation: CVH, cardiovascular health.

[a] All estimates are not accounted for complex survey design, due to participants without CVH data lack of the MEC exam weight.

[b] Other race includes American Indian or Alaska Native, Asian, Native Hawaiian or Pacific Islander, multiple races or ethnicities, or unknown.

© 2023 Mahemuti N et al. *JAMA Network Open.*

eTable 4. Adjusted Hazard Ratios and 95% CIs of CVH With Risk of All-Cause Mortality

		HR (95% CI)		
**Subgroup**	**Deaths (N)**	**Crude Model**	**Model 1[a]**	**Model 2[b]**
**CVH 80-100**	83 (3797)	Reference	Reference	Reference
**CVH 50-79**	872 (15686)	2.19 (1.70-2.82)	1.24 (0.95-1.61)	1.23 (0.94-1.60)
**CVH 0-49**	448 (4214)	4.75 (3.54-6.38)	1.77 (1.31-2.39)	1.71 (1.27-2.32)

Abbreviation: CVH, cardiovascular health; HR, hazard ratio; CI, confidence interval.

[a] Adjusted for age, sex, education level, poverty income ratio, marital status, race and ethnicity, estimated glomerular filtration rate.

[b] Adjusted for age; sex; education level; poverty income ratio; marital status; race and ethnicity; estimated glomerular filtration rate; and history of heart disease, stroke, and cancer.

© 2023 Mahemuti N et al. *JAMA Network Open.*

eTable 5. Adjusted Hazard Ratios and 95% CIs of UACR With Risk of All-Cause Mortality Stratified by Sex and CVH

Subgroup	Death(N)	HR (95%CI)[a]	*P* for trend
**Male**			
Per 10 mg/g increase	860 (11891)	1.41 (1.25-1.58)	-
CVH 80-100	46 (1577)	1.03 (0.59-1.81)	
CVH 50-79	554 (8113)	1.44 (1.28-1.63)	.008
CVH 0-49	260 (2201)	1.36 (1.04-1.78)	
**Female**			
Per 10 mg/g increase	543 (11806)	1.23 (1.05-1.44)	-
CVH 80-100	37 (2220)	0.77 (0.34-1.77)	
CVH 50-79	318 (7573)	1.18 (1.00-1.39)	.02
CVH 0-49	188 (2013)	1.48 (1.19-1.84)	

Abbreviation: CVH, cardiovascular health; UACR, Urinary albumin-to-creatinine ratio; HR, hazard ratio; CI, confidence interval.

[a] Adjusted for age; sex; education level; poverty income ratio; marital status; race and ethnicity; estimated glomerular filtration rate; and history of heart disease, stroke, and cancer.

© 2023 Mahemuti N et al. *JAMA Network Open.*

eTable 6. Adjusted Hazard Ratios and 95% CIs of UACR With Risk of All-Cause Mortality Stratified by Age and CVH

Subgroup	Death(N)	HR (95%CI)[a]	*P* for trend
**Age < 60 years**			
Per 10 mg/g increase	424 (17356)	1.33 (1.13-1.57)	-
CVH 80-100	30 (3275)	0.99 (0.51-1.94)	
CVH 50-79	242 (11369)	1.21 (1.00-1.47)	.03
CVH 0-49	152 (2712)	1.40 (1.07-1.84)	
**Age ≥ 60 years**			
Per 10 mg/g increase	979 (6341)	1.29 (1.16-1.42)	-
CVH 80-100	53 (522)	0.82 (0.44-1.53)	
CVH 50-79	630 (4317)	1.32 (1.18-1.48)	.003
CVH 0-49	296 (1502)	1.32 (1.07-1.64)	

Abbreviation: CVH, cardiovascular health; UACR, Urinary albumin-to-creatinine ratio; HR, hazard ratio; CI, confidence interval.

[a] Adjusted for age; sex; education level; poverty income ratio; marital status; race and ethnicity; estimated glomerular filtration rate; and history of heart disease, stroke, and cancer.

© 2023 Mahemuti N et al. *JAMA Network Open.*

eTable 7. Adjusted Hazard Ratios and 95% CIs of UACR With Risk of All-Cause Mortality Stratified by Hypertension and CVH

Subgroup	Death(N)	HR (95%CI)[a]	*P* for trend
**Hypertension**			
Per 10 mg/g increase	881 (8304)	1.24 (1.11-1.40)	-
CVH 80-100	25 (312)	0.88 (0.43-1.78)	
CVH 50-79	506 (5325)	1.22 (1.08-1.36)	.04
CVH 0-49	350 (2667)	1.32 (1.10-1.58)	
**Non-hypertension**			
Per 10 mg/g increase	522 (15393)	1.44 (1.20-1.72)	-
CVH 80-100	58 (3485)	0.87 (0.48-1.58)	
CVH 50-79	366 (10361)	1.46 (1.21-1.77)	.001
CVH 0-49	98 (1547)	1.64 (1.12-2.40)	

Abbreviation: CVH, cardiovascular health; UACR, Urinary albumin-to-creatinine ratio; HR, hazard ratio; CI, confidence interval.

[a] Adjusted for age; sex; education level; poverty income ratio; marital status; race and ethnicity; estimated glomerular filtration rate; and history of heart disease, stroke, and cancer.

© 2023 Mahemuti N et al. *JAMA Network Open.*

eTable 8. Adjusted Hazard Ratios and 95% CIs of UACR With Risk of All-Cause Mortality Stratified by Diabetes and CVH

Subgroup	Death(N)	HR (95%CI)[a]	*P* for trend
**Diabetes**			
Per 10 mg/g increase	337 (2847)	1.25 (1.07-1.47)	-
CVH 80-100	2 (59)	NA[b]	
CVH 50-79	162 (1581)	1.46 (1.21-1.77)	.004
CVH 0-49	173 (1207)	1.66 (1.12-2.40)	
**Non-diabetes**			
Per 10 mg/g increase	522 (20850)	1.31 (1.18-1.46)	-
CVH 80-100	81 (3738)	0.85 (0.53-1.37)	
CVH 50-79	710 (13395)	1.31 (1.17-1.46)	< .001
CVH 0-49	275 (2732)	1.45 (1.14-1.83)	

Abbreviation: CVH, cardiovascular health; UACR, Urinary albumin-to-creatinine ratio; HR, hazard ratio; CI, confidence interval.

[a] Adjusted for age; sex; education level; poverty income ratio; marital status; race and ethnicity; estimated glomerular filtration rate; and history of heart disease, stroke, and cancer.

[b] NA, not available for HR(95%CI) since failure to converge in modeling with limited sample size.

© 2023 Mahemuti N et al. *JAMA Network Open.*

eTable 9. Adjusted Hazard Ratios and 95% CIs of UACR With Risk of All-Cause Mortality in Models Further Adjusted for Covariates of CVH

Subgroup	Death (N)	HR (95%CI)[a]	*P* for trend
**CVH 80-100**	83 (3797)	0.87 (0.55-1.38)	
**CVH 50-79**	872 (15686)	1.32 (1.20-1.46)	< .001
**CVH 0-49**	448 (4214)	1.40 (1.17-1.68)	

Abbreviation: CVH, cardiovascular health; UACR, Urinary albumin-to-creatinine ratio; HR, hazard ratio; CI, confidence interval.

[a] Adjusted for age; sex; education level; poverty income ratio; marital status; race and ethnicity; estimated glomerular filtration rate; and history of heart disease, stroke, and cancer; HEI (continuous); minutes of moderate or greater intensity activity per week (continuous); nicotine exposure (current smoker, <1 quit/year, 1-5 quit/year, >5 quit/year, never smoker); average hours of sleep per night (continuous); body mass index (continuous); HbA1c (continuous); non–HDL cholesterol (continuous); systolic and diastolic BPs (continuous).

© 2023 Mahemuti N et al. *JAMA Network Open.*

eTable 10. Adjusted Hazard Ratios and 95% CIs of UACR With Risk of All-Cause Mortality Stratified by CVH After Exclusion of Participants Whose eGFR Was <60 mL/min/1.73 m^2

Subgroup	Death(N)	HR (95%CI)[a]	*P* for trend
**CVH 80-100**	69 (3722)	0.74 (0.43-1.28)	
**CVH 50-79**	720 (14971)	1.28 (1.14-1.45)	< .001
**CVH 0-49**	358 (3897)	1.41 (1.15-1.72)	

Abbreviation: CVH, cardiovascular health; UACR, Urinary albumin-to-creatinine ratio; HR, hazard ratio; CI, confidence interval; eGFR, estimated glomerular filtration rate.

[a] Adjusted for age; sex; education level; poverty income ratio; marital status; race and ethnicity; estimated glomerular filtration rate; and history of heart disease, stroke, and cancer.

© 2023 Mahemuti N et al. *JAMA Network Open.*

eTable 11. Adjusted Hazard Ratios and 95% CIs of UACR With Risk of All-Cause Mortality Stratified by CVH After Excluding Death Within the First 2 years of Follow-Up

Subgroup	Death(N)	HR (95%CI)[a]	*P* for trend
**CVH 80-100**	76 (3585)	1.08 (0.71-1.65)	
**CVH 50-79**	743 (14623)	1.28 (1.15-1.43)	< .001
**CVH 0-49**	392 (3824)	1.37 (1.18-1.58)	

Abbreviation: CVH, cardiovascular health; UACR, Urinary albumin-to-creatinine ratio; HR, hazard ratio; CI, confidence interval.

[a] Adjusted for age; sex; education level; poverty income ratio; marital status; race and ethnicity; estimated glomerular filtration rate; and history of heart disease, stroke, and cancer.

© 2023 Mahemuti N et al. *JAMA Network Open.*

eTable 12. Adjusted Hazard Ratios and 95% CIs of UACR With Risk of All-Cause Mortality Stratified by CVH With Additional Adjustment for Hypertension and Diabetes

Subgroup	Death(N)	HR (95%CI)[a]	*P* for trend
**CVH 80-100**	83 (3797)	0.91 (0.59-1.40)	
**CVH 50-79**	872 (15686)	1.31 (1.19-1.45)	< .001
**CVH 0-49**	448 (4214)	1.38 (1.16-1.63)	

Abbreviation: CVH, cardiovascular health; UACR, Urinary albumin-to-creatinine ratio; HR, hazard ratio; CI, confidence interval.

[a] Adjusted for age; sex; education level; poverty income ratio; marital status; race and ethnicity; estimated glomerular filtration rate; and history of heart disease, stroke, and cancer.

© 2023 Mahemuti N et al. *JAMA Network Open.*

eTable 13. Estimated Direct and Indirect Effect Sizes of Cardiovascular Health With All-Cause Mortality Through UACR in the National Health and Nutrition Examination Survey, 2005 to 2018

Subgroup	Deaths (N)	HR (95% CI) [a]			
		Total Effect	Direct Effect	Indirect Effect	% Mediated
**CVH 80-100**	83 (3797)	Reference	Reference	Reference	Reference
**CVH 50-79**	872 (15686)	1.34 (1.21-1.50)	1.32 (1.18-1.47)	1.02 (1.01-1.03)	7.8
**CVH 0-49**	448 (4214)	1.82 (1.48-2.24)	1.74 (1.41-2.15)	1.05 (1.03-1.07)	10.5

Abbreviation: CVH, cardiovascular health; UACR, Urinary albumin-to-creatinine ratio; HR, hazard ratio; CI, confidence interval.

[a] Adjusted for age; sex; education level; poverty income ratio; marital status; race and ethnicity; estimated glomerular filtration rate; and history of heart disease, stroke, and cancer.

© 2023 Mahemuti N et al. *JAMA Network Open.*

eFigure 1. Selection of Study Participants From the 2005 to 2018 Cycles of the National Health and Nutrition Examination Survey

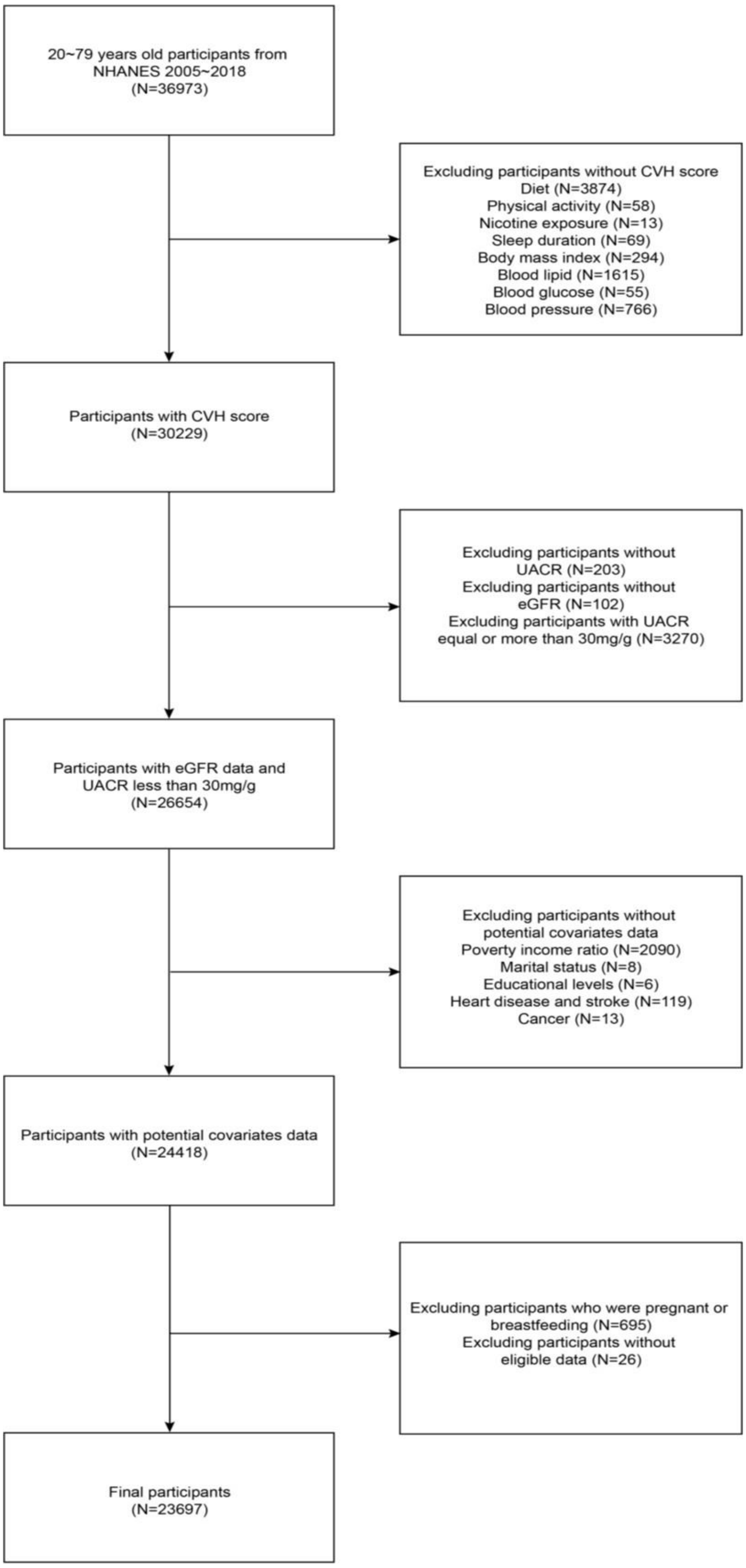

© 2023 Mahemuti N et al. *JAMA Network Open.*

eFigure 2. HRs (95% CIs) for Associations of UACR With All-Cause Mortality Stratified by CVH Groups, After Adjusting for Hypertension and Diabetes

The multivariable Cox regression model adjusted for age; sex; race and ethnicity; education level; marital status; poverty income ratio; estimated glomerular filtration rate; and history of heart disease, stroke, cancer; hypertension; and diabetes. CVH, cardiovascular health; HR, hazard ratio; UACR, urinary albumin-to-creatinine ratio.

Subgroup	Death(N)	HR (95% CI)	*P* for trend
**CVH 80-100**			
UACR low	21 (1388)	Reference	
UACR medium	28 (1284)	0.75 (0.41-1.35)	
UACR high	34 (1125)	1.13 (0.62-2.07)	
**CVH 50-79**			
UACR low	187 (4973)	0.91 (0.58-1.44)	
UACR medium	240 (5079)	1.24 (0.76-2.01)	<.001
UACR high	445 (5634)	1.45 (0.92-2.28)	
**CVH 0-49**			
UACR low	79 (975)	1.49 (0.89-2.49)	
UACR medium	104 (1241)	1.61 (0.96-2.70)	
UACR high	265 (1998)	2.02 (1.25-3.25)	

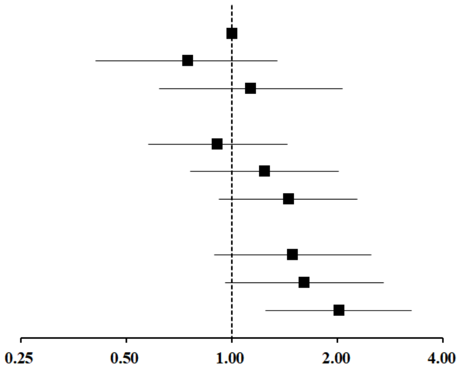

© 2023 Mahemuti N et al. *JAMA Network Open.*

eReferences

1. Food patterns equivalents database: methodology and user guides. Agricultural Research Service, US Department of Agriculture. Accessed March 1, 2023. https://www.ars.usda.gov/northeast-area/beltsville-md-bhnrc/beltsville-human-nutrition-research-center/food-surveys-research-group/docs/fped-methodology/

2. Lloyd-Jones DM, Allen NB, Anderson CAM, et al. Life's Essential 8: Updating and Enhancing the American Heart Association's Construct of Cardiovascular Health: A Presidential Advisory From the American Heart Association. *Circulation*. 2022;146(5):e18-e43. doi:10.1161/cir.0000000000001078

© 2023 Mahemuti N et al. *JAMA Network Open.*
