# Supplementary material for: Urinary Albumin-to-Creatinine Ratio in Normal Range, Cardiovascular Health, and All-Cause Mortality
Source: JAMA Netw Open. 2023 Dec 19;6(12):e2348333. doi: 10.1001/jamanetworkopen.2023.48333 (PMC10731498; doi:10.1001/jamanetworkopen.2023.48333)
Supplement: Supplement 2. — Data Sharing Statement [file jamanetwopen-e2348333-s002.pdf]

Data Sharing Statement

Mahemuti. Urinary Albumin-to-Creatinine Ratio in Normal Range, Cardiovascular Health, and All-Cause Mortality. *JAMA Netw Open*. Published December 19, 2023. doi:10.1001/jamanetworkopen.2023.48333

Data
Data available: No
